# Proteomic Analysis of Caspofungin-Induced Responses in Planktonic Cells and Biofilms of *Candida albicans*

**DOI:** 10.3389/fmicb.2021.639123

**Published:** 2021-02-18

**Authors:** Peng Li, Chaminda J. Seneviratne, Qingxian Luan, Lijian Jin

**Affiliations:** ^1^Department of Periodontology, Peking University School and Hospital of Stomatology, Beijing, China; ^2^Division of Periodontology and Implant Dentistry, Faculty of Dentistry, The University of Hong Kong, Hong Kong SAR, China; ^3^National Dental Research Institute Singapore, National Dental Centre Singapore, Singapore, Singapore

**Keywords:** *Candida albicans*, caspofungin, biofilms, antifungal resistance, proteomics

## Abstract

*Candida albicans* biofilms display markedly increased antifungal resistance, and the underlying mechanisms remain unclear. This study investigated the signature profiles of *C. albicans* planktonic cells and biofilms in response to caspofungin (CAS) by mass spectrometry-based shotgun proteomics. We found that *C. albicans* biofilms were twofold more resistant to CAS with reference to planktonic cells. Notably, 9.6% of *C. albicans* biofilm cells survived the lethal treatment of CAS (128 μg/ml), confirmed by LIVE/DEAD staining, confocal laser scanning microscopy (CLSM) and scanning electron microscopy analyses. The responses of *C. albicans* planktonic cells and biofilms to CAS treatment at respective minimum inhibitory concentrations (MICs) were assessed by high-throughput proteomics and bioinformatics approaches. There were 148 and 224 proteins with >twofold difference identified from the planktonic cells and biofilms, respectively. CAS treatment downregulated several cell wall- and oxidative stress-related proteins. Whereas, CAS-induced action was compensated by markedly increased expression of many other proteins involved in cell wall integrity and stress response (e.g., heat shock proteins). Moreover, considerable expression changes were identified in metabolism-associated proteins like glycolysis, tricarboxylic acid (TCA) cycle and ATP biosynthesis. Importantly, various key proteins for cell wall integrity, stress response and metabolic regulation (e.g., PIL1, LSP1, HSP90, ICL1, and MLS1) were exclusively enriched and implicated in *C. albicans* biofilms. This study demonstrates that *C. albicans* biofilms undergo highly complicated yet complex regulation of multiple cellular pathways in response to CAS. Signature proteins essential for modulating cell wall integrity, stress response and metabolic activities may account for the antifungal resistance of *C. albicans* biofilms.

## Introduction

*Candida albicans* remains the predominant pathogen for various refractory superficial and systemic infections with high morbidity and mortality (Seneviratne et al., 2015; Lohse et al., 2018). It is a frequent colonizer on tissue surfaces and implanted medical devices, forming adherent biofilms capable of withstanding currently limited arsenal of antifungals. It has been well documented that *C. albicans* biofilms display markedly increased antifungal resistance with reference to the planktonic counterparts, whereas the underlying mechanisms are not fully understood (Taff et al., 2013). The resilient nature of biofilms makes the management of biofilm-related infections extremely difficult. A thorough understanding of the resistance mechanisms of *C. albicans* biofilms is thus of great importance for development of novel anti-biofilm strategies and approaches.

The echinocandins represent a novel class of antifungals that exert fungicidal activity by noncompetitively inhibiting β-1,3-glucan synthase required for fungal cell wall biosynthesis (Denning, 2003). Inhibition of this enzyme results in depletion of cell wall β-glucans and cell lysis. Caspofungin (CAS) as the first licensed echinocandin has been widely used to treat invasive candidiasis with high efficacy and low toxicity. Clinical resistance to the echinocandins is uncommon, despite occurrence of resistance in some isolates of *Candida* species, ranging 2–13% for *Candida glabrata* and <3% for *C. albicans* as well as most *Candida* species (Perlin, 2015; Larkin et al., 2018; Coste et al., 2020). Among the antifungals approved for clinical application, echinocandins and amphotericin B show consistent effects against *Candida* biofilms of susceptible species (Bujdakova, 2016). Nevertheless, *Candida* biofilms-induced infections frequently account for therapeutic failure (Andes et al., 2012). Of note, *C. albicans* biofilms are 2–20 times more resistant to CAS as compared to its planktonic form (Tobudic et al., 2010; Taff et al., 2013).

Proteomics offers valuable information on global protein expression, and thus has emerged as an important tool for uncovering molecular mechanisms underlying complex biological phenomena. In particular, mass spectrometry-based shotgun proteomics has been widely used to investigate antimicrobial resistance (Park et al., 2016). Our recent proteomic study has demonstrated that delicate metabolic control and synchronized stress response crucially account for the antifungal tolerance of *Candida* biofilm persisters to amphotericin B (Li et al., 2015). It is known that certain proteins are differently expressed upon transition from planktonic growth to biofilm formation, and the unique protein expression profiles in biofilms may contain valuable insights into drug resistance (Hall and Mah, 2017; Seneviratne et al., 2020). It is therefore intriguing to hypothesize that *Candida* biofilms exert specific protein expression different from planktonic cells under antifungal treatment, thereby allowing biofilms to exhibit highly increased antifungal resistance. However, there are limited data on antifungal-induced proteomic changes in *C. albicans* biofilms. This study investigated the signature profiles of *C. albicans* planktonic cells and biofilms in response to CAS exposure via mass spectrometry-based shotgun proteomics.

## Materials and Methods

### Culture and Growth of *C. albicans*

*Candida albicans* BF-1, a well-characterized clinical strain for its biofilm-producing capacity in previous reports (Seneviratne et al., 2008; Li et al., 2015; Krishnamoorthy et al., 2020), was selected for this study. The yeast was subcultured on Sabouraud dextrose agar (SDA; Gibco Ltd., Paisley, United Kingdom) at 37°C for 24 h, and inoculated into yeast nitrogen base (YNB; Difco, Franklin Lakes, NJ, United States) medium containing 50 mM glucose in an orbital shaker at 80 rpm. The cells were harvested after overnight culture, washed twice using PBS, and then re-suspended in YNB medium supplemented with 100 mM glucose and adjusted to 1 × 10^7^ CFU/ml, prior to use for biofilm formation. For proteomic analysis, the standardized suspension was further cultured for 48 h before antifungal treatment.

### Formation of Biofilms

*Candida albicans* biofilms were grown on the polystyrene surface of 24-well plates (Thermo Fisher Scientific) unless otherwise specified. Briefly, 1 ml of the standardized suspension (1 × 10^7^ CFU/ml) was aliquoted into the wells and allowed to adhere for 1.5 h at 37°C in a shaker at 80 rpm. Afterward, cell suspensions were aspirated, and loosely adherent cells were removed by washing with PBS. The wells were replenished with 1 ml of YNB medium containing 100 mM glucose and the plates were incubated for 48 h at 37°C in a shaker at 80 rpm.

### Antifungal Susceptibility Testing and XTT Assay

Caspofungin obtained from Merck & Co., Inc. (Rahway, NJ, United States) was used in the experiments. Stock solutions of CAS (6.4 mg/ml) were prepared in sterile water. The susceptibility of planktonic cells was determined by broth microdilution method according to CLSI document M27-A3 (CLSI, 2008). *C. albicans* inoculum suspensions and twofold serial dilutions of CAS were made in RPMI 1640 medium (Life Technologies, New York, NY, United States) and dispensed into 96-well plates, yielding an inoculum density of 0.5 × 10^3^–2.5 × 10^3^ CFU/ml and drug concentrations of 0.03–16 μg/ml. Minimum inhibitory concentration (MIC) endpoints were determined after 24 h incubation to be the lowest concentration of drug with a prominent decrease in turbidity.

The MIC of *C. albicans* biofilms was determined by XTT reduction assay as shown previously (Li et al., 2015). An XTT (0.2 mg/ml)-menadione (4 μM) (Sigma-Aldrich) solution was prepared fresh in PBS. *C. albicans* biofilms were formed and then treated with 0.25–128 μg/ml of CAS in 1 ml of YNB medium containing 100 mM glucose for 24 h at 37°C. The biofilms were washed twice with PBS, and 1 ml of XTT-menadione solution was added to each pre-washed well of the plates. The plates were incubated in the dark for 3 h at 37°C. Afterward, 200 μl of the supernatant was transferred to a 96-well plate, and the absorbance was detected in a SpectraMAX 340 Tunable Microplate Reader (Molecular Devices Ltd., Sunnyvale, CA, United States) at 490 nm. The MIC value was calculated on the basis of a 50% reduction in metabolic activity referring to the drug-free control. The experiments were performed on three separate occasions to determine the MICs of planktonic cells and biofilms.

### Quantitation of Biofilms

*Candida albicans* biofilms were treated with CAS (0.25–256 μg/ml) for 24 h and then washed twice with PBS. The biofilms were collected by scrapping and vigorous vortexing in 1 ml of PBS. The CFU of live cells were counted via serial dilution and plating on SDA, and the total cell number of untreated biofilms was calculated as well.

### Scanning Electron Microscopy

*Candida albicans* biofilms formation was made on Thermanox^TM^ Coverslips (Thermo Fisher Scientific), and they were treated with CAS (0.5, 1, and 128 μg/ml) for 24 h. The samples were rinsed with PBS, placed in fixative (2% glutaraldehyde) for 2 h, and then dehydrated in a graded series of ethanol (70% for 10 min, 95% for 10 min, 100% for 20 min) and air dried in a desiccator. Subsequently, the specimens were coated with platinum/palladium and visualized in a scanning electron microscopy (SEM) system (Hitachi SU-1510, Tokyo, Japan).

### LIVE/DEAD Staining

The viability of CAS-treated biofilms was further evaluated by LIVE/DEAD staining and confocal laser scanning microscopy (CLSM). *C. albicans* biofilms were established on ibiTreat μ-Slide 8 well (ibidi, Martinsried, Germany) and treated with CAS (0.5, 1 and 128 μg/ml). After 24 h exposure, the biofilms were washed twice with PBS and stained with LIVE/DEAD BacLight Viability kit (Molecular Probes, Eugene, OR, United States) in dark for 30 min. Images were captured by scanning the biofilms in FLUOVIEW FV 1000 CLSM system (Olympus, Tokyo, Japan).

### Proteomic Profiling

*Candida albicans* biofilms and age-matched planktonic cells cultured in YNB medium containing 100 mM glucose were treated with CAS at concentrations equivalent to their respective MICs for 24 h. The CAS-exposed cells and untreated controls were washed twice with PBS and collected for protein extraction. The cell pellets from three independent experiments were lysed with Y-PER^TM^ Yeast Protein Extraction Reagent (Pierce Biotechnology, Rockford, IL, United States). After centrifugation at 13,200 rpm for 10 min, the supernatants were collected and protein concentration was examined by Bradford assay (Bio-Rad, Hercules, CA, United States). The prepared samples were subsequently assessed by liquid chromatography-tandem mass spectrometry (LC-MS/MS) with a nanoflow high-performance liquid chromatography (HPLC) coupled to an LTQ-Orbitrap Velos mass spectrometer (Thermo Fisher Scientific) as previously described (Li et al., 2018).

### Data Processing and Statistical Analysis

The mass spectrometry proteomics data were uploaded to ProteomeXchange with identifier-PXD023480 via the iProX partner repository (Ma et al., 2019). Raw data were analyzed with the MaxQuant software (Version 1.5.0.25) (Cox and Mann, 2008). MS/MS spectra were searched with the Andromeda search engine against the database of the reference strain *C. albicans* SC5314 (UniProt release 2015-05). The parameters for protein identification and label-free quantification were set as previously described (Li et al., 2018). For first and main searches, the peptide mass tolerance was set at 20 ppm and 4.5 ppm, respectively. Methionine oxidation and N-terminal acetylation were adopted as variable modification, and carbamidomethylation as fixed modification. The minimum peptide length was 7 amino acids, allowing a maximum of 2 missed cleavages. A false discovery rate (FDR) of 0.01 was set for identification of peptides and proteins. If multiple proteins were identified from the same set of peptides, a protein group was presented. Statistical analysis was performed by *t*-test with a cut-off permutation-based FDR value of 0.05 using the Perseus software (Version 1.5.0.9) (Cox and Mann, 2012). Hierarchical clustering was applied to group proteins based on their expression profiles. The significant proteins were subjected to further gene ontology (GO) enrichment analysis using BiNGO (Version 3.0.3) (Maere et al., 2005). A GO database for *C. albicans* SC5314 obtained from EMBL-EBI (2015_08) was used to annotate the significant proteins. Corrections of multiple hypothesis testing were performed with a Benjamini-Hochberg FDR threshold of 0.05.

## Results

### *Candida albicans* Susceptibility to CAS

**FIGURE 1:**
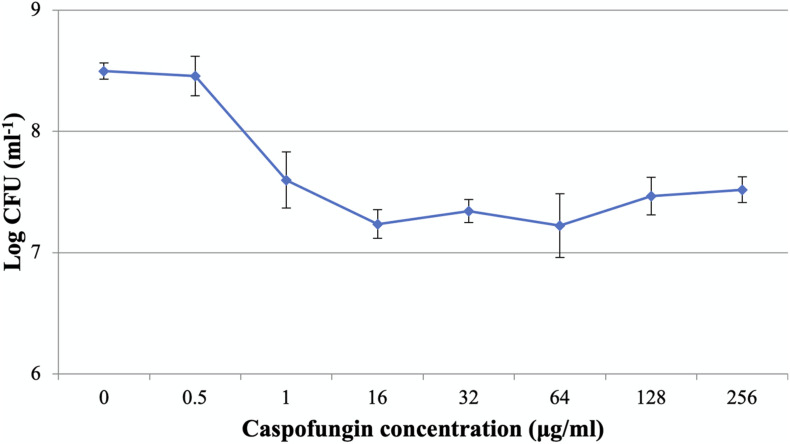
Caspofungin-induced inhibition of *C. albicans* biofilms for 24 h. The increase of CAS concentration failed to eradicate the biofilms. Data represent means ± SD of three biological replicates from one representative experiment out of two independent ones with similar results.

The MICs of CAS against *C. albicans* planktonic cells and biofilms were 0.5 and 1 μg/ml, respectively. The dose-dependent treatment by CAS resulted in two distinct subpopulations of *C. albicans* biofilms (Figure 1). As CAS concentration increased, the number of viable cells maintained at a similar level. Over 90% of the biofilm cells were killed, while 9.6% of cells survived even at a high concentration of CAS (128 μg/ml) toxic to host cells.

### Visualization of CAS-Treated *C. albicans* Biofilms

**FIGURE 2:**
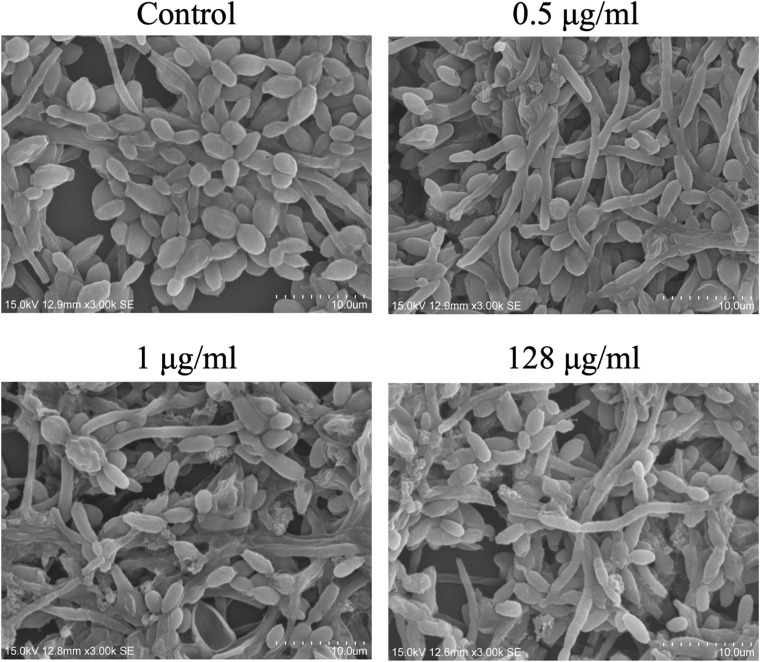
Scanning electron microscopy images of CAS-treated *C. albicans* biofilms for 24 h. One representative field out of two independent ones is presented. Scale bar = 10 μm.

**FIGURE 3:**
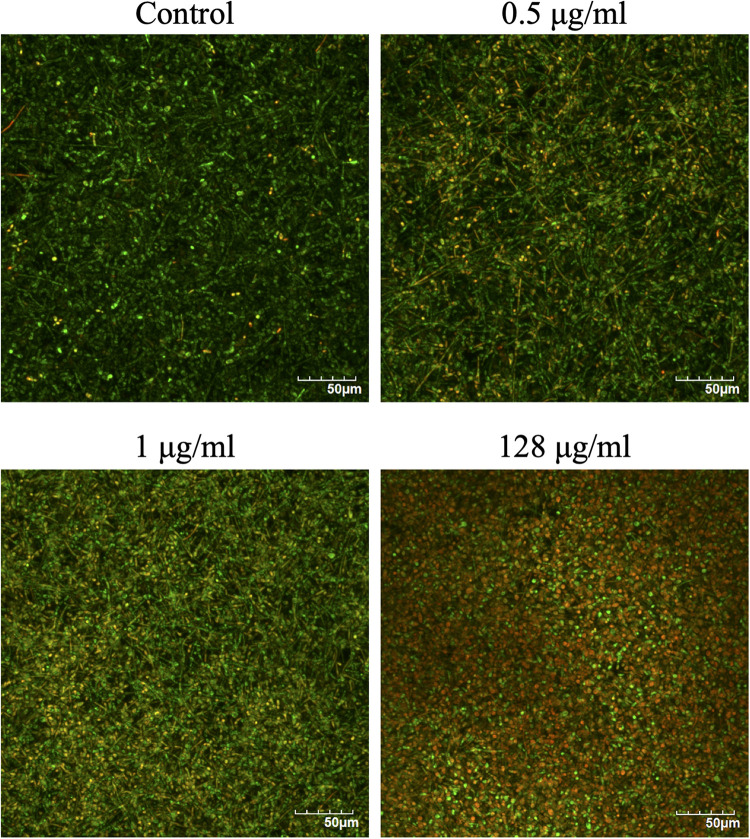
Confocal laser scanning microscopy images of CAS-treated *C. albicans* biofilms for 24 h. The biofilms were stained with viability indicators (SYTO 9 and propidium iodide). Live cells stain green and dead cells are red/yellow-colored. One representative experiment from two independent ones is shown. Scale bar = 50 μm.

The effects of CAS exposure on *C. albicans* biofilms at different concentrations were further examined by SEM and CLSM. Three working concentrations were selected, including sub-MIC (0.5 μg/ml), MIC (1 μg/ml) and a high dose (128 μg/ml). The architecture of untreated *C. albicans* biofilms was highly heterogeneous, consisting of a mixture of yeasts, pseudohyphae and hyphae (Figures 2, 3).

The viability of CAS-treated *C. albicans* biofilms was analyzed by LIVE/DEAD staining (Figure 3). The increase of CAS concentration failed to control the biofilms, confirming the presence of CAS-tolerant cells. The SEM images revealed that many biofilm cells were collapsed, and they displayed aberrant morphologies after CAS treatments (Figure 2). When exposed to inhibitory concentrations of CAS (1 and 128 μg/ml), a fraction of cells appeared unaffected by the treatment with comparable morphology to the controls.

### Altered Protein Expression Profiles of *C. albicans* Planktonic Cells and Biofilms in Response to CAS

**FIGURE 4:**
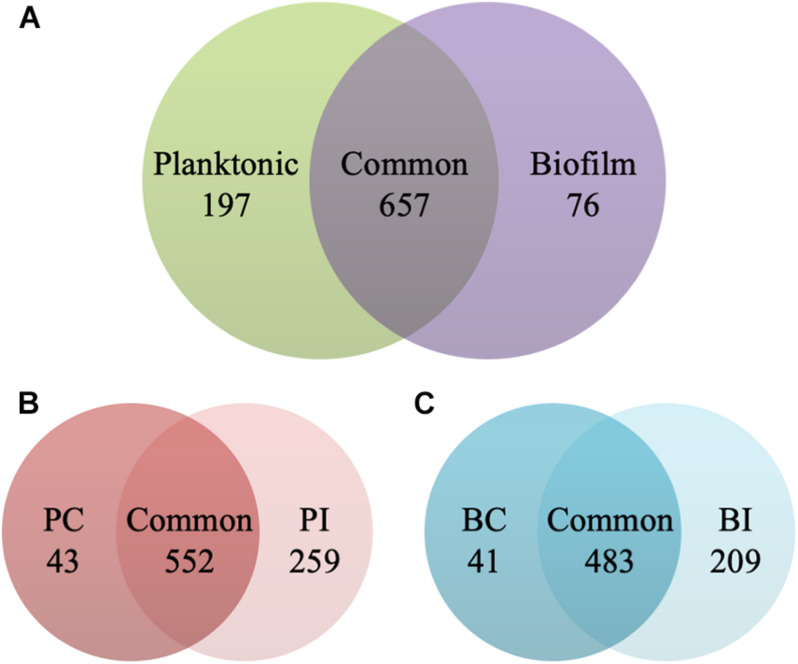
Identification of proteins in the planktonic cells and biofilms of *C. albicans*. **(A)** Overall distribution of total protein/protein groups present in all control and CAS-treated planktonic cells and biofilms of *C. albicans*. **(B)** Distribution of proteins identified in control (PC) and CAS-treated (PI) planktonic cells of *C. albicans*. **(C)** Distribution of proteins identified in control (BC) and CAS-treated (BI) biofilms of *C. albicans*.

The planktonic cells and biofilms of *C. albicans* were exposed to 0.5 and 1 μg/ml CAS for 24 h, respectively. The samples analyzed in the experiments were highly reproducible with average Pearson correlation coefficient values of 0.907 for the control and 0.950 for the CAS-treated planktonic samples, respectively. The correlation coefficient for the control and CAS-treated biofilm samples was 0.924 and 0.873, respectively. Overall, a total of 930 proteins/protein groups were identified from all control and CAS-treated planktonic and biofilm samples, and of them 854 were present in planktonic cells and 733 in biofilm cells with 657 in common (Figure 4A). Of those 854 proteins identified in planktonic cells, 595 were detected in controls and 811 in the CAS-treated cells with 552 in common (Figure 4B). Of those 733 proteins identified in biofilm cells, 524 were detected in controls and 692 in CAS-treated cells with 483 in common (Figure 4C).

**FIGURE 5:**
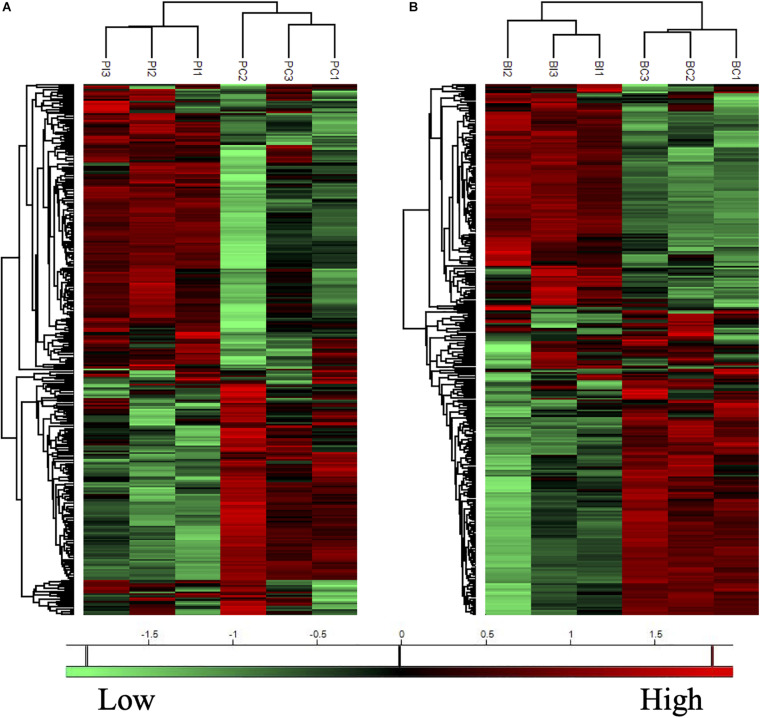
Heatmaps of protein expression profiles of the planktonic cells **(A)** and biofilms **(B)** of *C. albicans* with or without CAS treatment. Treated (PI and BI) and untreated (PC and BC) samples are grouped separately in both planktonic cells and biofilms of *C. albicans*. Proteins with similar expression patterns are clustered in the left column trees. Scale bar depicts relative intensity.

**FIGURE 6:**
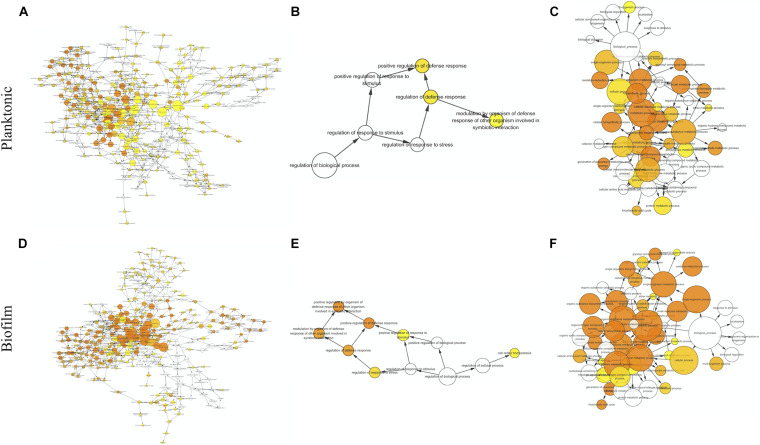
Biological processes identified by gene ontology enrichment analysis in the planktonic cells **(A–C)** and biofilms **(D–F)** of *C. albicans*. Relationship networks for all the identified biological processes **(A,D)**, and those associated with stress response **(B,E)** and cellular metabolism **(C,F)**. Yellow nodes represent significantly enriched GO biological processes. For more significant *p* values, the node color is increasingly more orange. Uncolored nodes are not overrepresented.

The proteins with valid intensity values in all three biological replicates under each condition were subjected to hierarchical clustering and statistical analyses, highlighting 332 and 310 eligible proteins in planktonic cells and biofilms respectively (Figure 5). The statistical analysis indicated 148 and 224 significantly altered proteins (permutation-based FDR, 0.05) with >twofold difference from the CAS-exposed planktonic cells and biofilms with reference to their untreated controls, respectively (Supplementary Datasets 1, 2). GO enrichment analysis revealed highly intricated interlinked networks of biological processes involved in the responses of *C. albicans* planktonic cells (Figure 6A) and biofilms (Figure 6D) to CAS. The annotated differentially expressed proteins are listed in Supplementary Table 1.

### Pathways Involved in the Responses of *C. albicans* Planktonic Cells to CAS

Among the 148 proteins with significant differences in the CAS-treated planktonic cells, the abundance of 77 proteins increased and that of 71 proteins decreased. In the planktonic cells exposed to CAS, various significant proteins involved in stress response and cell wall maintenance were identified (Figure 6B and Supplementary Table 1). The expression of oxidative stress response-associated proteins decreased, such as TRX1, TTR1 and GPS2. In addition, the treatment downregulated certain cell wall-related proteins (MP65, SIM1, AGM1, PMI1, BGL2, and XOG1). By contrast, there was increased expression of proteins involved in cell wall integrity (MPG1, RHO1, GFA1, and ERG11) and heat shock response (HSP78, HSP104, BMH1, and TPS2) as well as other stress proteins (e.g., TUP1, FAS1, and ARF2).

A series of CAS-responsive proteins were implicated in cellular metabolic pathways (Figure 6C and Supplementary Table 1). The proteins associated with glycolysis were mainly downregulated, such as two regulatory enzymes (HXK2 and PGK1). Whereas, a key enzyme (PFK2) catalyzing the rate-limiting step of glycolysis was slightly upregulated. The CAS treatment suppressed the expression of proteins involved in pentose phosphate pathway, and induced higher level of proteins responsible for pyruvate metabolism. Except MDH1/2, the majority of enzymes from tricarboxylic acid (TCA) cycle were upregulated like IDH2 and SDH1/2. The abundance of proteins required for electron/proton transport and ATP synthesis (e.g., ATP1/2/3) was elevated. Furthermore, a number of ribosomal proteins were enriched in the CAS-treated planktonic cells.

### Pathways Involved in the Responses of *C. albicans* Biofilms to CAS

The significant hits in the CAS-exposed biofilms included 126 upregulated- and 138 downregulated-proteins. Similar to the planktonic cells, the CAS-treated biofilms showed considerable changes in diverse proteins associated with stress response and cellular metabolism (Figures 6E,F and Supplementary Table 1). The CAS treatment inhibited the expression of various proteins involved in oxidative stress response (e.g., TRX1, TTR1, GPS2, and SOD2) and cell wall maintenance (MP65, SIM1, AGM1, PMI1, and BGL2). Interestingly, a few proteins important for antioxidative response (TSA1, HSP21, and LPD1) and cell wall integrity (MPG1, RHO1, PIL1, LSP1, PSA2, and CSP37) were upregulated. Moreover, there were overexpressed heat shock proteins (HSP21, HSP60, HSP78, HSP90, HSP104, and BMH1) and other stress-responsive proteins (e.g., TUP1, FAS1, MSI3, SSB1, KAR2, GRP2, and CSH1).

The expression of most of the proteins (e.g., HXK2, PGK1, and GPM1) involved in glycolysis decreased, whereas that of the rate-limiting enzyme phosphofructokinase (PFK1) greatly increased. Reduced expression of proteins involved in pentose phosphate pathway and overexpression of those responsible for pyruvate metabolism were observed. The expression of major enzymes associated with TCA cycle (e.g., IDH2, KGD1, and SDH1) was activated, while that of a few ones (e.g., MDH1/2 and IDP1/2) was inhibited. Furthermore, the CAS-treated biofilms expressed higher levels of proteins involved in electron/proton transport and ATP synthesis (e.g., ATP1/2/3/5/7), and various ribosomal proteins were also identified with increased abundance.

Of note, proteins linked to several metabolic pathways were primarily regulated in the CAS-treated biofilms but not in the planktonic cells (Supplementary Table 1). Overexpression was observed in the key enzymes accounting for glyoxylate cycle including ICL1 and MLS1. Besides the upregulation of PDB1 which activates the common way of acetyl-CoA generation, the expression of ACS2 catalyzing the alternative reaction for synthesis of acetyl-CoA was enhanced. A variety of proteins associated with amino acid biosynthesis were differentially expressed, with the majority of them downregulated. Several elongation factors required for translational elongation expressed to higher levels, especially CEF3.

## Discussion

The ability of *Candida* biofilms to withstand antifungals represents a critical risk for therapeutic failure and recurrent infections. In this study, antifungal susceptibility testing demonstrated that *C. albicans* biofilms were more resistant to CAS with reference to the planktonic counterparts. Dose-dependent treatment of *C. albicans* biofilms revealed a biphasic pattern, and the increase of CAS concentration failed to eradicate the biofilms. A high level of viable cells (9.6%) could survive the CAS treatment at 128 μg/ml, indicating that CAS has a limited effect on *C. albicans* biofilms in consistence with a previous observation (LaFleur et al., 2006). Given the susceptibility of planktonic cells to CAS, a large fraction of biofilm survivors that otherwise would be killed may significantly contribute to the increased CAS resistance in the *C. albicans* biofilms. Indeed, these biofilms were well established under the culture conditions as visualized with CLSM and SEM. The decrease of hyphal cells in the biofilms exposed to 128 μg/ml of CAS suggests that CAS may suppress the pathogenicity and invasiveness of biofilms. However, the biofilms remained attached after treatment, and obviously eradication of them failed, demonstrating their high resistance and tolerance to CAS.

Current evidence suggests that *Candida* biofilm resistance is complex and multifactorial involving redundant and integrated mechanisms such as altered metabolism, extracellular matrix, stress response and presence of persisters (Taff et al., 2013). In this study, comparative shotgun proteomic analysis was performed to investigate the responses of *C. albicans* planktonic cells and biofilms treated by CAS. A total of 930 proteins were identified with a powerful hybrid LTQ-Orbitrap instrument which features high speed, sensitivity, resolution and mass accuracy (Kalli et al., 2013). The present experiment outputted nearly four times as many proteins as a previous gel-based proteomic analysis of CAS-treated *C. albicans* planktonic cells (Hoehamer et al., 2010). Among the 41 proteins listed in that study, 38 proteins were identified in our study, and around 60% of them were similarly regulated, such as RHO1, ATP2 and IDH2. After exposure to CAS for 24 h, both the planktonic cells and biofilms exhibited significant changes of proteomic profiles in comparison to the untreated controls. Notably, there are almost twofold more responsive proteins in the biofilms than the planktonic cells upon exposure to CAS, suggesting biofilms invoke a more complex response against CAS.

Caspofungin disrupts cell wall integrity by inhibiting the synthesis of its major constituent β-1,3-glucan. The CAS treatment inhibited the expression of several proteins required for cell wall maintenance (e.g., SIM1, AGM1, and PMI1) in both the planktonic cells and biofilms, indicative of the compromised integrity of cell wall. In the CAS-treated planktonic cells, there was reduced expression of BGL2 encoding a glucan transferase and XOG1 encoding an exoglucanase that are responsible for glucan modification and extracellular matrix glucan delivery (Taff et al., 2012). Although both would not affect cell wall concentration of β-1,3-glucan (Taff et al., 2012), inhibition of these enzymes may result from its decreased production. Interestingly, there was increased expression of proteins responsible for glycosylation (MPG1), chitin formation (GFA1), and ergosterol biosynthesis (ERG11) essential for cell wall integrity. In comparison to the planktonic cells, the CAS-treated biofilms exhibited much higher expression of MPG1, GFA1 and ERG11. *C. albicans* cell wall is primarily composed of β-glucans, chitin and mannoproteins. Elevated chitin biosynthesis is linked with reduced susceptibility to echinocandins both *in vitro* and *in vivo* (Perlin, 2015). Indeed, increased chitin content of cell wall has been observed during the paradoxical growth of *Candida* species, representing a rescue mechanism against high levels of echinocandins (Stevens et al., 2004; Melo et al., 2007; Rueda et al., 2014; Toth et al., 2020). Additionally, ergosterol is a major constituent of the fungal plasma membrane (Dupont et al., 2012). The upregulation of MPG1, GFA1, and ERG11 in the present study suggests that the impaired cell wall due to CAS treatment is strengthened by incorporation of major cell wall/membrane components. A GTPase serving as a key regulator of β-1,3-glucan synthase, RHO1 (Santos et al., 2003), was greatly upregulated in the biofilms and only slightly induced in the planktonic cells in response to CAS. The activation of RHO1 is relevant to the action mechanism of CAS and consistent with the need for glucan synthesis. Besides, overexpression of other cell wall biosynthesis-related proteins like PIL1, LSP1, PSA2, and CSP37 occurred in the CAS-treated biofilms but not in the planktonic cells. It has been suggested that both PIL1 and LSP1 associate with β-1,3-glucan synthase along with RHO1, and serve as important sphingolipid-dependent regulators of cell wall integrity signaling (Edlind and Katiyar, 2004). Therefore, *C. albicans* responds to cell wall-disrupting CAS by induction of the proteins involved in cell wall maintenance, and its biofilms appear to exhibit increased resistance to cell wall stress with reference to the planktonic cells.

In the presence of CAS, many proteins associated with antioxidative response were repressed in both planktonic cells and biofilms, such as TRX1 and TTR1. While, a few antioxidants (TSA1, HSP21, and LPD1) were induced in the CAS-treated biofilms, suggesting that biofilms may partially maintain oxidative stress response. Nevertheless, there is lack of evidence on the induction of oxidative damage in *Candida* biofilms by CAS (Delattin et al., 2014). This may decrease the need for adaptation to oxidative stress, and suggests that antioxidative capacity may play a limited role in the resistance of *C. albicans* biofilms to CAS. Meanwhile, CAS treatment enhanced the expression of several proteins involved in heat shock response (e.g., FAS1 and HSP104) in both planktonic cells and biofilms. FAS1 encodes fatty acid synthase that is important for generation and maintenance of cell membranes, thereby contributing to survival of diverse microorganisms (Nguyen et al., 2012). HSP104 is required for adaptation to thermal stress and virulence in *C. albicans* (Fiori et al., 2012). Notably, more stress proteins are involved in the response of biofilms to CAS, such as MSI3, CSH1, GRP2, SSB1, KAR2 HSP21, HSP60, and HSP90. Among them, MSI3 and CSH1 have been associated with resistance to antifungal azoles (Schulz et al., 2011; Nagao et al., 2012). GRP2 is linked with the response to oxidative and osmotic stresses (Karababa et al., 2004). The rest of the proteins belong to heat shock protein family critical for stress response (Santoro, 2000; Mayer et al., 2012; Ramirez-Quijas et al., 2015). HSP90 could facilitate the development of antifungal resistance in *C. albicans* and *Saccharomyces cerevisiae* (Cowen and Lindquist, 2005), and actually it governs echinocandin and azole resistance in *C. albicans* via activation of calcineurin (Singh et al., 2009). Collectively, these findings suggest that *C. albicans* biofilms enable activation of strong stress response that crucially contributes to biofilm resistance.

An array of proteins from pyruvate metabolic process, TCA cycle and ATP biosynthesis were markedly overexpressed in the CAS-exposed biofilms, in agreement with the hypothesis that enhanced energy demand is required for stress adaptation during CAS treatment (Hoehamer et al., 2010). The planktonic cells responded similarly, but only around half number of proteins were mapped with relatively smaller changes in abundance. However, glycolysis is likely to be inhibited in both CAS-treated planktonic cells and biofilms. A range of ribosomal proteins were enriched in both conditions in line with a transcription analysis on CAS-treated *C. albicans* biofilms exhibiting overexpressed genes involved in ribosome biogenesis (Vediyappan et al., 2010). The upregulated ribosomes may be required for metabolic regulation and synthesis of essential proteins under a stressed condition. Furthermore, the biofilms involve specific metabolic pathways in response to CAS. It is noted that the glyoxylate cycle, an alternative pathway of TCA cycle, is associated with *Candida* virulence, and its inhibition results in growth reduction of *C. albicans* (Lorenz and Fink, 2001; Cheah et al., 2014). The activation of this pathway in the biofilms may increase their viability and account for biofilm resistance. The downregulation of multiple proteins associated with amino acid biosynthesis implies that the *C. albicans* biofilms may shut down unnecessary protein synthesis, while several elongation factors with enhanced expression function in coordination with the ribosomal proteins.

## Conclusion

The present study demonstrates that *C. albicans* biofilms are strongly resistant to CAS by formation of a subfraction of CAS-tolerant cells. To our knowledge, this is the first comparative analysis of proteomic signatures of CAS-treated *C. albicans* planktonic cells and biofilms. Our findings indicate that *C. albicans* biofilms enable to undergo highly complicated yet complex regulation of multiple cellular pathways with reference to the planktonic cells in response to CAS treatment. The expression of signature proteins essential for modulating cell wall integrity, stress response and metabolic activities may account for the antifungal resistance of *C. albicans* biofilms.

## Data Availability Statement

The datasets presented in this study can be found in online repositories. The names of the repository/repositories and accession number(s) can be found below: ProteomeXchange Consortium; PXD023480.

## Author Contributions

PL and LJ conceived and designed the study. PL performed the experiments. PL, CS, and LJ analyzed the data. PL, CS, QL, and LJ wrote the manuscript. All authors contributed to the article and approved the submitted version.

## Conflict of Interest

The authors declare that the research was conducted in the absence of any commercial or financial relationships that could be construed as a potential conflict of interest.
