# Supplementary material for: Proteomic Analysis of Caspofungin-Induced Responses in Planktonic Cells and Biofilms of Candida albicans
Source: Front Microbiol. 2021 Feb 18;12:639123. doi: 10.3389/fmicb.2021.639123 (PMC7931687; doi:10.3389/fmicb.2021.639123)
Supplement: Supplementary file 3 [file Table_1.docx]

**﻿SUPPLEMENTAL TABLE 1** Selected annotated differentially expressed proteins in CAS-treated planktonic cells and biofilms of *C. albicans*

| **UniProt accession no.** | **Protein description** | **Gene** | **Log_2_ fold change** | | | |
| --- | --- | --- | --- | --- | --- | --- |
|  |  |  | **Planktonic cells** | | **Biofilms** | |
| **Glycolysis** | | | | | |  |
| Q9URB4 | Fructose-bisphosphate aldolase | FBA1 | **-1.09** | **-1.55** | |  |
| P46273 | Phosphoglycerate kinase | PGK1 | **-1.55** | **-2.03** | |  |
| P83776 | Hexokinase-2 | HXK2 | **-1.37** | **-2.12** | |  |
| Q9P940 | Triosephosphate isomerase | TPI1 | **-1.95** | **-2.10** | |  |
| Q5AGZ8 | ATP-dependent 6-phosphofructokinase | PFK2 | **1.11** | 3.26 N/A | |  |
| Q5AK53 | ATP-dependent 6-phosphofructokinase | PFK1 | 1.67 N/S | **4.40** | |  |
| P82612 | Phosphoglycerate mutase | GPM1 | -0.52 N/S | **-1.68** | |  |
| P30575 | Enolase 1 | ENO1 | -0.50 N/S | **-1.30** | |  |
| **Pentose-phosphate pathway** | | | | | |  |
| Q5A017 | Transaldolase | TAL1 | **-1.44** | **-2.40** | |  |
| Q59PZ6 | 6-phosphogluconolactonase | SOL3 | **-3.05** | **-3.79** | |  |
| **Tricarboxylic acid cycle** | | | | | |  |
| Q59ZZ5 | Citrate synthase | CIT1 | **1.27** | **1.69** | |  |
| Q5AMP4 | Malate dehydrogenase | MDH1-1 | **-1.63** | **-2.35** | |  |
| P83778 | Malate dehydrogenase, cytoplasmic | MDH1 | **-1.82** | **-2.82** | |  |
| Q5A0T8*^a^* | Isocitrate dehydrogenase [NAD] subunit, mitochondrial | IDH2 | **1.99** | **2.57** | |  |
| Q5A0M1*^a^* | Isocitrate dehydrogenase [NAD] subunit, mitochondrial | IDH2 |  |  |  |  |
| Q5A1E8 | Succinate dehydrogenase flavoprotein subunit, mitochondrial | SDH12 | **1.27** | **1.73** | |  |
| Q59QN7 | Succinate dehydrogenase iron-sulfur subunit, mitochondrial | SDH2 | **1.73** | 2.17 N/A | |  |
| Q59V07*^a^* | Isocitrate dehydrogenase [NADP] | IDP2 | -0.28 N/S | **-1.47** | |  |
| Q59V43*^a^* | Uncharacterized protein | IDP2 |  |  |  |  |
| Q59MF7 | Isocitrate dehydrogenase [NADP] | IDP1 | -0.26 N/S | **-1.23** | |  |
| P82611 | Aconitate hydratase, mitochondrial | ACO1 | -0.61 N/S | **-1.14** | |  |
| Q59LN7 | 2-oxoglutarate dehydrogenase, mitochondrial | KGD1 | 1.65 N/A | **4.94** | |  |
| **Glyoxylate cycle** | | | | | |  |
| Q59RB8 | Isocitrate lyase | ICL1 | -0.29 N/A | **3.27** | |  |
| Q5APD2 | Malate synthase | MLS1 | 0.51 N/A | **2.72** | |  |
| **Pyruvate metabolic process** | | | | | |  |
| Q5AGX8 | Acetyltransferase component of pyruvate dehydrogenase complex | LAT1 | **2.13** | **3.98** | |  |
| Q5A5V6 | Pyruvate dehydrogenase E1 component subunit beta, mitochondrial | PDB1 | **1.90** | **3.43** | |  |
| Q5AHK8 | Likely pyruate carboxylase | PYC2 | **1.58** | **3.73** | |  |
| Q5A0Z9 | Pyruvate dehydrogenase E1 component subunit alpha | PDA1 | 1.40 N/S | **3.68** | |  |
| P83779 | Pyruvate decarboxylase | PDC11 | 0.75 N/S | **1.20** | |  |
| Q8NJN3 | Acetyl-coenzyme A synthetase 2 | ACS2 | 0.56 N/S | **1.16** | |  |
| **ATP synthesis** | | | | | |  |
| Q59RA8*^a^* | ATP synthase subunit alpha | ATP1 | **1.29** | **2.85** | |  |
| Q59RD8*^a^* | ATP synthase subunit alpha |  |  |  |  |  |
| Q59UR7 | ATP synthase subunit beta | ATP2 | **1.25** | **2.66** | |  |
| Q5A8A4 | ATP synthase subunit gamma | ATP3 | **1.09** | **2.32** | |  |
| Q59ZE0 | F-type H+-transporting ATPase subunit B | ATP4 | **1.54** | **3.20** | |  |
| Q5APK5 | Cytochrome c oxidase subunit 4 | COX5 | **1.64** | **3.51** | |  |
| Q59PT0 | V-type proton ATPase subunit B | VMA2 | **1.72** | **3.43** | |  |
| Q5AJB1 | V-type proton ATPase catalytic subunit A | TFP1 | **2.08** | 3.82 N/A | |  |
| P53698 | Cytochrome c | CYC1 | **-2.06** | 0.35 N/S | |  |
| Q5A7P7 | ATP synthase F1, delta subunit | ATP5 | 0.86 N/A | **2.65** | |  |
| Q59PV8 | F-type H+-transporting ATPase subunit D | ATP7 | 0.19 N/A | **2.13** | |  |
| Q5ABS1 | Cytochrome b-c1 complex subunit 7 | QCR7 | 0.41 N/A | **1.34** | |  |
| P83782 | Cytochrome b-c1 complex subunit 2, mitochondrial | QCR2 | 1.34 N/S | **2.12** | |  |
| **Cell wall integrity** | | | | | |  |
| Q59XX2 | Cell surface mannoprotein MP65 | MP65 | **-2.05** | **-2.36** | |  |
| Q5AKU5 | Secreted beta-glucosidase SIM1 | SIM1 | **-2.75** | **-2.25** | |  |
| P34948 | Mannose-6-phosphate isomerase | PMI1 | **-1.94** | **-2.56** | |  |
| Q5AKW4 | Phosphoacetylglucosamine mutase | AGM1 | **-2.24** | **-3.25** | |  |
| Q5AMT2 | Glucan 1,3-beta-glucosidase BGL2 | BGL2 | **-2.01** | **-1.88** | |  |
| O93827 | Mannose-1-phosphate guanyltransferase | MPG1 | **1.39** | **2.26** | |  |
| O42825 | GTP-binding protein RHO1 | RHO1 | **1.04** | **1.77** | |  |
| P29717 | Glucan 1,3-beta-glucosidase | XOG1 | **-1.46** | -1.17 N/A | |  |
| P53704 | Glutamine--fructose-6-phosphate aminotransferase | GFA1 | **1.95** | 3.62 N/A | |  |
| P10613 | Lanosterol 14-alpha demethylase | ERG11 | **1.65** | 3.07 N/A | |  |
| Q5AL34 | Mannose-1-phosphate guanylyltransferase | PSA2 | 0.76 N/S | **1.42** | |  |
| Q5A9D4 | Cell surface protein | CSP37 | 0.75 N/S | **1.36** | |  |
| Q59VF6 | Possible sphingolipid long chain base sensory protein | PIL1 | 0.73 N/S | **1.30** | |  |
| Q59KV8 | Possible sphingolipid long chain base sensory protein | LSP1 | 0.73 N/S | **1.26** | |  |
| **Response to oxidative stress** | | | | | |  |
| Q5A915 | Thioredoxin | TXL1 | **-2.54** | **-3.13** | |  |
| Q5ACN1 | Thioredoxin | TRX1 | **-3.70** | **-5.04** | |  |
| Q5AD39 | Peptide-methionine (S)-S-oxide reductase | MXR1 | **-2.17** | **-2.59** | |  |
| Q5ABB1 | Glutaredoxin | TTR1 | **-3.31** | **-2.75** | |  |
| Q59WW7 | Glutathione peroxidase | GPS2 | **-1.31** | **-2.12** | |  |
| Q59WC0 | Superoxide dismutase 1 copper chaperone | LYS7 | **-1.37** | **-1.18** | |  |
| Q5A8Z4 | Superoxide dismutase | SOD2 | -0.61 N/S | **-1.57** | |  |
| Q5AG89 | Thioredoxin reductase | TRR1 | -0.78 N/S | **-1.32** | |  |
| Q9Y7F0 | Peroxiredoxin TSA1 | TSA1 | 0.88 N/S | **2.09** | |  |
| Q5AEN1 | Cytochrome c peroxidase, mitochondrial | CCP1 | -0.22 N/S | **-1.05** | |  |
| Q59RQ6 | Dihydrolipoyl dehydrogenase | LPD1 | 0.97 N/S | **1.21** | |  |
| **Response to heat** | | | | | |  |
| Q5A376 | Uncharacterized protein | HSP104 | **1.58** | **3.21** | |  |
| O42766 | 14-3-3 protein homolog | BMH1 | **1.10** | **2.37** | |  |
| Q96UX5 | Heat shock protein 78, mitochondrial | HSP78 | **1.97** | **4.38** | |  |
| Q5AI14 | Trehalose-phosphatase | TPS2 | **1.48** | 1.90 N/A | |  |
| Q5AHH4 | Small heat shock protein 21 | HSP21 | 1.29 N/S | **2.76** | |  |
| P46598 | Heat shock protein 90 homolog | HSP90 | 1.12 N/S | **3.15** | |  |
| O74261 | Heat shock protein 60, mitochondrial | HSP60 | 1.14 N/S | **3.02** | |  |
| **Other stress/pathogenesis proteins** | | | | | |  |
| P83774 | Guanine nucleotide-binding protein subunit beta-like protein | ASC1 | **1.21** | **2.46** | |  |
| P0CY34 | Transcriptional repressor TUP1 | TUP1 | **1.12** | **1.30** | |  |
| Q5ADT4 | Uncharacterized protein | GCY1 | **-2.49** | **-2.79** | |  |
| Q5A4W7 | Fatty acid synthase subunit beta | FAS1 | **2.27** | **4.57** | |  |
| Q5AND9 | ADP-ribosylation factor 1 | ARF2 | **1.19** | 2.97 N/A | |  |
| Q59L12 | Agglutinin-like protein 3 | ALS3 | -3.29 N/A | **-2.22** | |  |
| Q5A940 | Multiprotein-bridging factor 1 | MBF1 | -1.54 N/S | **-2.78** | |  |
| Q59L90 | Uncharacterized protein | HET1 | 0.58 N/A | **1.57** | |  |
| Q59QH2 | Uncharacterized protein | CSH1 | 1.33 N/S | **2.75** | |  |
| Q5A397 | Hsp75-like protein | SSB1 | 0.93 N/S | **1.04** | |  |
| Q96VB9 | Heat shock protein homolog SSE1 | MSI3 | 0.86 N/S | **2.22** | |  |
| Q5AD54 | Likely HSP70/BiP chaperone | KAR2 | 0.89 N/S | **1.27** | |  |
| P83775 | Putative NADPH-dependent methylglyoxal reductase GRP2 | GRP2 | 0.62 N/S | **1.85** | |  |
| **Translational elongation** | | | | | |  |
| Q59QS2 | Potential translation elongation factor Cam1p | CAM1-1 | **1.31** | **2.38** | |  |
| Q59K68*^a^* | Elongation factor 1-alpha | TEF1 | 0.63 N/S | **1.02** | |  |
| P0CY35*^a^* | Elongation factor 1-alpha 1 | TEF1 |  |  |  |  |
| Q59QD6*^a^* | Elongation factor 1-alpha 2 | TEF2 |  |  |  |  |
| P25997 | Elongation factor 3 | CEF3 | 1.96 N/S | **4.96** | |  |
| **Cellular amino acid biosynthetic process** | | | | | |  |
| Q5A3K7 | D-3-phosphoglycerate dehydrogenase 1 | SER33 | **1.64** | **3.65** | |  |
| Q59TC4 | Homocitrate synthase, mitochondrial | LYS22 | **1.87** | **4.42** | |  |
| Q5A0E3 | Cysteine synthase | CYS4 | -1.04 N/S | **-2.13** | |  |
| Q59P52 | Phosphoserine aminotransferase | SER1 | -0.03 N/S | **-1.20** | |  |
| Q5A9D9 | Homoisocitrate dehydrogenase, mitochondrial | LYS12 | -0.26 N/S | **-1.02** | |  |
| P0CY20 | 3'(2'),5'-bisphosphate nucleotidase 1 | HAL21 | -0.30 N/A | **-2.40** | |  |
| Q59YS9 | Branched-chain-amino-acid aminotransferase | BAT22 | -0.73 N/S | **-1.83** | |  |
| Q59SM8 | Likely C1-tetrahydrofolate synthase | MIS11 | 1.13 N/S | **1.46** | |  |
| Q5A644 | Homoaconitase, mitochondrial | LYS4 | 0.17 N/S | **-1.50** | |  |
| Q5AGE6 | Uncharacterized protein | THR4 | -0.91 N/S | **-1.92** | |  |
| P79023 | Phospho-2-dehydro-3-deoxyheptonate aldolase, tyrosine-inhibited | ARO4 | 0.03 N/S | **-1.52** | |  |
| Q5AFI8 | 3-isopropylmalate dehydrogenase | LEU2 | -0.77 N/S | **-2.21** | |  |
| Q5AIA2 | Homoserine dehydrogenase | HOM6 | -0.59 N/S | **-1.80** | |  |
| P82610 | 5-methyltetrahydropteroyltriglutamate--homocysteine methyltransferase | MET6 | -0.90 N/S | **-1.85** | |  |
| **Ribosome** | | | | | |  |
| Q59N00 | Likely cytosolic ribosomal protein S3 | RPS3 | **2.04** | **5.02** | |  |
| Q59LS1 | 60S ribosomal protein L3 | RPL3 | **2.38** | **3.49** | |  |
| Q9UVJ4 | 60S ribosomal protein L10a | RPL10A | **1.89** | **2.72** | |  |
| Q5A8Y0*^a^* | 40S ribosomal protein S4 | RPS42 | **2.06** | **4.26** | |  |
| Q5A5Q8*^a^* | 40S ribosomal protein S4 | RPS4A |  |  |  |  |
| Q5AJ93 | 40S ribosomal protein S7-A | RPS7A | **1.51** | **2.93** | |  |
| Q5A389 | 40S ribosomal protein S20 | RPS20 | **1.98** | **2.52** | |  |
| Q5AML4*^a^* | 60S ribosomal protein L20 | RPL20B | **1.53** | **4.14** | |  |
| Q5AN19*^a^* | 60S ribosomal protein L20 | RPL20B |  |  |  |  |
| Q5A109 | Polyubiquitin | UBI3 | **-2.63** | **-1.71** | |  |
| Q5AJF7 | 60S ribosomal protein L12 | RPL12 | **1.54** | **3.05** | |  |
| Q5A7K0 | 40S ribosomal protein S24 | RPS24 | **2.40** | **4.24** | |  |
| Q59ZX4 | 60S ribosomal protein L4-B | RPL4B | **1.30** | **2.39** | |  |
| Q5AFQ0 | Likely cytosolic ribosomal protein S18 | RPS18 | **2.00** | **4.65** | |  |
| Q5AG43 | 40S ribosomal protein | RPS5 | **1.66** | **4.04** | |  |
| Q5ADQ6 | 40S ribosomal protein S12 | RPS12 | **1.56** | **3.14** | |  |
| O42817 | 40S ribosomal protein S0 | RPS0 | **1.63** | **4.40** | |  |
| P40910 | 40S ribosomal protein S1 | RPS1 | **1.68** | **4.37** | |  |
| Q96W54 | 40S ribosomal protein S22 | RPS22A | **1.51** | **3.38** | |  |
| Q5AMI6 | 40S ribosomal protein S6 | RPS6A | **1.58** | **2.87** | |  |
| Q59T44 | 40S ribosomal protein S8 | RPS8A | **2.10** | 2.62 N/A | |  |
| Q5ANC2 | Likely cytosolic ribosomal protein L18 | RPL18 | **2.60** | 3.94 N/A | |  |
| Q5ALV6 | 40S ribosomal protein S26-B | RPS26A | **1.52** | 2.97 N/A | |  |
| Q5AHF6 | Likely cytosolic ribosomal protein L21 | RPL21A | **1.23** | 2.25 N/A | |  |
| Q5ANH6 | Likely cytosolic ribosomal protein S15 | RPS15 | **1.82** | N/A | |  |
| Q5AB87 | 60S ribosomal protein L16-A | RPL16A | **2.18** | 4.17 N/A | |  |
| Q5A6A1 | 60S ribosomal protein L24 | RPL24A | **1.80** | 3.05 N/A | |  |
| Q5AGZ7 | 60S ribosomal protein L5 | RPL5 | 1.17 N/S | **1.53** | |  |
| Q5AEN2 | Likely cytosolic ribosomal protein L9 | RPL9B | 1.63 N/S | **4.32** | |  |
| O59931 | 60S ribosomal protein L13 | RPL13 | 1.57 N/S | **2.71** | |  |
| Q59WJ0 | Likely cytosolic ribosomal protein L8 | RPL82 | 1.23 N/S | **2.50** | |  |
| Q59M73 | 60S ribosomal protein L23 | RPL23A | 2.14 N/A | **3.86** | |  |
| Q5AFQ4 | 60S acidic ribosomal protein P0 | RPP0 | 1.78 N/S | **3.95** | |  |
| Q59Z66 | Likely cytosolic ribosomal protein L11 | RPL11 | 1.06 N/S | **2.42** | |  |

*^a^* Protein group;

N/S, Not significant;

N/A, Not available for statistical analysis due to the absence of protein identification in certain sample(s).
